# Rational decision making in medicine: Implications for overuse and underuse

**DOI:** 10.1111/jep.12851

**Published:** 2017-12-01

**Authors:** Benjamin Djulbegovic, Shira Elqayam, William Dale

**Affiliations:** ^1^ Department of Supportive Care Medicine City of Hope Duarte California USA; ^2^ Department of Hematology City of Hope, Duarte California USA; ^3^ School of Applied Social Sciences, Division of Psychology De Montfort University Leicester UK

**Keywords:** clinical decision making, health policy, overtreatment, overuse, practice, rationality, undertreatment, underuse

## Abstract

In spite of substantial spending and resource utilization, today's health care remains characterized by poor outcomes, largely due to overuse (overtesting/overtreatment) or underuse (undertesting/undertreatment) of health services. To a significant extent, this is a consequence of low‐quality decision making that appears to violate various rationality criteria. Such suboptimal decision making is considered a leading cause of death and is responsible for more than 80% of health expenses. In this paper, we address the issue of overuse or underuse of health care interventions from the perspective of rational choice theory. We show that what is considered rational under one decision theory may not be considered rational under a different theory. We posit that the questions and concerns regarding both underuse and overuse have to be addressed within a specific theoretical framework. The applicable rationality criterion, and thus the “appropriateness” of health care delivery choices, depends on theory selection that is appropriate to specific clinical situations. We provide a number of illustrations showing how the choice of theoretical framework influences both our policy and individual decision making. We also highlight the practical implications of our analysis for the current efforts to measure the quality of care and link such measurements to the financing of health care services.

## 1 INTRODUCTION

It is no secret that today's health care system is in crisis^1^, ^2^: Societies devote a substantial amount of resources to health care, and yet patient outcomes remain inferior. The United States alone spends nearly 18% ($3.2 trillion) of its gross domestic product on health care; however, only 55% of needed services are delivered and more than 30% is inappropriate and, therefore wasteful, “care.”^3^ Ultimately, the observed (suboptimal) care relates to the quality of medical decisions.^3^ Indeed, it has been contended that personal decisions are the leading cause of death^4^ and that physicians' decisions are responsible for 80% of health care expenditures.^5^, ^6^ If decision making can largely explain the relatively poor state of affairs of current health care utilization, the logical question to ask is as follows: Are the decisions made during doctor‐patient encounters, in fact, rational? In a recent paper, we reviewed existing theories of rationality and their implications for medical practice.^7^ We found that no single model of rationality can fit all medical contexts; what is considered “rational behaviour” under one rationality theory may be considered “irrational” under another one.^7^ We call this “normative pluralism,” which, as explained in detail below, calls for the matching of a given clinical situation/problem with a given theory of rationality.

In this paper, we extend this analysis of rational decision making in clinical medicine to demonstrate the practical importance of this debate for the question of overuse (overtesting/overtreatment) and underuse (undertesting/undertreatment). We show that theory choice determines the “rational” course of action, both at the level of individual and policy decision making.

## 2 BRIEF OVERVIEW OF PRINCIPLES AND THEORIES OF RATIONALITY

Rationality is commonly defined as decision making that helps us achieve our *goals*.^8^, ^9^ In the context of clinical medicine, this typically means the desire to improve our health. Rationality does not guarantee that a decision is error free; rather, rational decision making accounts for the potential consequences of possible errors of our action—false negatives and false positives—to help us arrive at optimal outcomes. Theories of rationality for decision making are broadly classified as *descriptive* theories (which depict how people *actually* make their decisions) and *normative* ones (addressing the question how people “should” or “ought to” make their decisions). In‐between are *prescriptive* theories, which prescribe routes of action expected to be effective given what is known about human cognitive processes and cognitive architecture.^10^ Table 1 displays a short summary description of some of the most common theories of rationality relevant to clinical medicine. Table 2 provides an overview of the core ingredients that are common across most theoretical constructs of rationality.^7^ We next illustrate the issues of overuse and underuse in medicine that can be observed under each of these theories. *Overuse* refers to “too much care” and is defined as “provision of a service that is unlikely to increase the quality or quantity of life, that poses more harm than benefit, or that patients who were fully informed of its potential benefits and harms would not have wanted”; *underuse* refers to “too little care,” defined as “failure to deliver a service that is highly likely to improve the quality or quantity of life, that represents good value for the money, and that patients who were fully informed of its potential benefits and harms would have wanted.”^51^ Thus, both overuse and underuse are defined relative to the goals of the decision maker—in this case, a fully informed patient and his or her physician.

**Table 1:** A list of major theories and models of rationality relevant to medical decision making^7^

**Figure 1:**
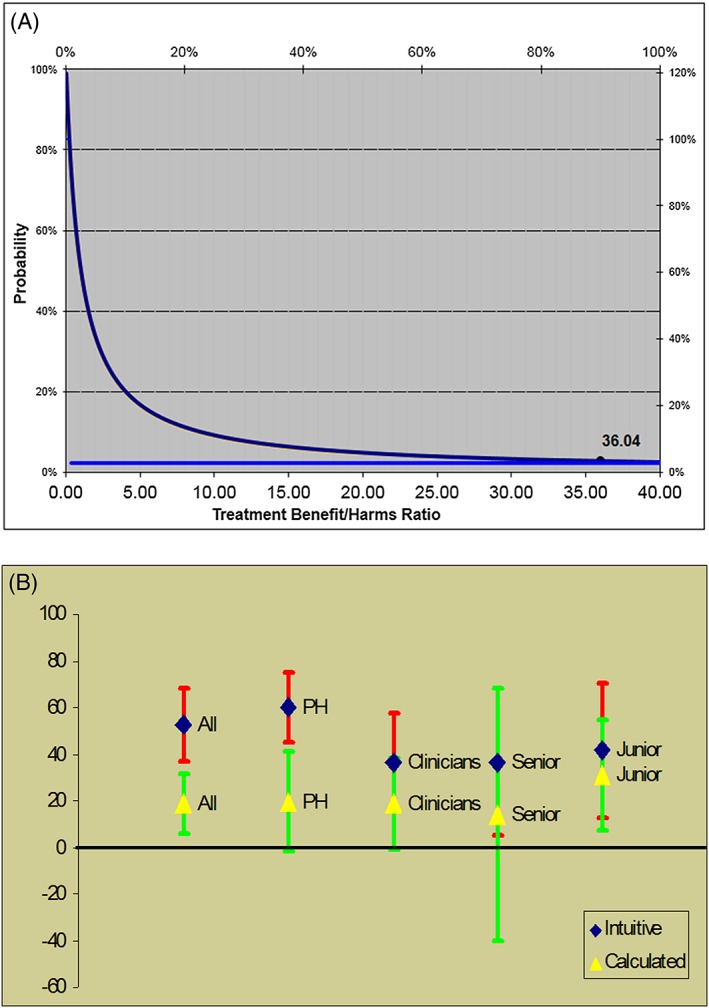
Threshold model of decision making. A, The model states that the most rational decision is to prescribe treatment when the expected treatment benefit outweighs its expected harms at given probability of disease or clinical outcome. The horizontal line indicates the probability at which physicians should treat the patient with suspected tuberculosis (2.7%). B, Actual threshold for treating a patient suspected of having tuberculosis (based on Basinga et al^50^; graph: Courtesy of Dr Jef Van den Ende (see Table 1 and text for details)

Normative theories of rationality
**Evidence‐based medicine approach to rational decision making** 11, 12: A normative theory, which posits that there is a link between rationality and believing what is true. (Our actions and beliefs are justifiable [or reasonable/rational] as a function of the trustworthiness of the evidence, and the extent to which we believe that evidence is determined by credible processes.) See also Epistemic rationality
Example: Clinical practice guidelines panels more readily recommend health interventions if the quality of evidence supporting such a recommendation is high.^13^
**Epistemic rationality**: The rationality based on acquisition of true/fit‐for‐purpose knowledge. Linked to new mind rationality^14^ (see also Grounded rationality).
Example: Evidence‐based medicine approach to decision making.
**Expected utility theory (EUT)—decision analysis/Bayesian rationality** 15: The type of rationality associated with conformity to a normative standard such as the probability calculus or classical logic. In medicine, the most dominant normative theory is EUT, which is based on mathematical axioms of rationality, according to which rational choice is associated with selection of the alternative with higher expected utility (expected utility is the average of all possible results weighted by their corresponding probabilities). It is typically based on Bayesian probability calculus.
Example: Decision analyses such as EUT‐based microsimulation model to develop screening recommendations for colorectal cancer.^16^
Descriptive theories of rationality
**Pragmatic/instrumental rationality/rationality**_**1**_ 8 **or substantive rationality** 17, 18: A descriptive theory, which proposes that rationality depends on the content and not only on the structure of decisions (process), and that the content should be assessed in light of short‐ and long‐term goals (purpose). Fits with the descriptivist approach,^19^ which argues that empirical evidence cannot support the “oughtness” of a model.
**Adaptive or ecological rationality** 20, 21: A variant of bounded rationality, which stipulates that human decision making depends on the context and environmental cues; hence, rational behaviour/decision making requires adaptation to environment/patient circumstances. Sometimes referred to as “Panglossian,”^9^, ^22^ the position that humans should be considered to be a priori rational due to optimal evolutionary processes.
Example: Extrapolation of research evidence to specific patient circumstances including social context and co‐morbidities dominates medical practice.
**Argumentative Theory of Reasoning** 23, 24 proposes that reason and rational thinking has evolutionary evolved with primary social function to justify one's self and convince others to believe one and gain their trust.
Example: Doctors invoke evidence‐based knowledge out of sense that it would be approved by the medical community and, in doing so, preserve their reputation and improve the health of their patients.
**Bounded rationality** 25, 26: Posits that, reflective of the principle that rationality should respect epistemological, environmental, and computational constraints of human brains, rational behaviour relies on s*atisficing* process (finding a good enough solution) instead of EUT maximizing approach. The *heuristic approach* to decision making is the mechanism of implementation of bounded rationality.^27^ Often linked to prescriptive models of rationality^28^ designs for improvement of human rationality informed by cognitive architecture.
Example: Simple fast‐and‐frugal tree using readily available clinical cues outperformed 50 variables multivariable logistic model regarding decision whether to admit the patient with chest pain to coronary care unit.^21^
**Deontic introduction theory** 29: A descriptive theory of inference from “is” to “ought”, which implies that rationality requires integration of the evidence related to the problem at hand (“is”) with the goals and values to decisions and actions (“ought”), while taking context into account. See also Grounded rationality.
Example: Evidence (“is”) shows that if prostate cancer patients receive detailed information about hormone therapy, their decision making style improves; policymakers infer that patients should receive detailed information.^30^
**Dual processing theories of rational thought (DPTRT)** 9: A family of theories based on the architecture of human cognition, contrasting intuitive (“type 1”) processes with effortful (“type 2”) processes. A descriptive variant of this approach is that the rational action should be coherent with formal principles of rationality as well as human intuitions about good decisions. The normative/prescriptive variant of this theory is sometimes referred to as “Meliorism,”^9^, ^22^ the position that humans are often irrational but can be educated to be rational. According to Meliorist principles, when the goals of the genes clash with the goals of the individual (see below), the rational course of action should be dictated by the latter.
Example: Physicians often adjust their recommendations based on their intuition.^31^
DPTRT can be thought of as a combination/contrast of
**Old mind/evolutionary rationality/rationality of the genes** 14, 32: The rationality linked to evolutionarily instilled goals (sex, hunger, etc). Past oriented and relying on type 1 mechanisms, it is driven by the evolutionary past and by experiential learning
Example: Eating chocolates when one has to reduce weight.
**New mind/individual rationality** 14, 32: The rationality linked to the goals of the individual rather than those of the genes. It is future oriented and relies on type 2 mechanisms, most importantly the ability to run mental simulations of future events and hypothetical situations. This is what enables humans to think consequentially and solve novel problems
Example: Use of contraceptives. The genes' goal is to self‐replicate, ie, to produce more copies of themselves. Contraceptives negate this goal while allowing humans greater individual freedom.
**Grounded rationality** 33: A descriptive theory, which postulates that rationality should be judged within epistemic context (ie, what is known to a decision maker and his or her goals), and that rational course of action is the one that facilitates the achievement of our goals given the context. See also Pragmatic rationality.
Example: To achieve health goals, physicians typically recommend treatment with which they are familiar/know about.
**Meta‐rationality** 34 **or the master rationality motive** 35: Relies on DPTRT and posits that rationality represents hierarchical goal integration while taking into account both emotions and reasons. It also refers to integration of so‐called *Thin theories of rationality*: theories in which the goals, context, and desires of behaviour are not evaluated (as per, for example, applying EUT without taking patient's desires into account)—that is, any goal is as good as any other goal—with *Broad theories of rationality*: theories of rationality in which the goals and desires of the decision‐maker are evaluated within context and in such a way as to achieve hierarchical coherence within goals.^32^, ^36^
Example: Meta‐rationality model of rationality subsumes other variants of DPTRT. The approach based on meta‐rationality is often characteristic of a “wise” physician; the approach is particularly evident in high‐stake, high‐emotional decisions such as end‐of‐life where the substantive goals about achievable health status have to be reconciled with patient/physician emotional reaction to a proposed decision
Example: Pragmatic rationality dominates clinical decision making particularly in the fields such as oncology, where desirable health goals (eg, cure) may not be possible; as a result, the re‐evaluation of both goals and decision procedures may be needed (eg, switch from aggressive treatment to palliative care in advanced incurable cancers)
**Regret regulation‐rationality is characterized by regulation of regret** 37: This is a variant of DPTRT that relies on regret, which as a cognitive emotion uses counterfactual reasoning processes to tap into the analytical aspect of our cognitive architecture as well as into affect‐based decision making. According to this view, medical rational decision making is associated with regret‐averse decision processes.
Example: Contemporary medical practice has increasingly adopted the practice that patients' values and preferences should be consulted before a given health intervention is given. Patient values and preferences heavily depend on emotions such as regret, which, if properly elicited, may improve vigilance in decision making. 38, 39, 40
**Robust satisficing** 17, 18: A variant of regret‐based DPTRT, according to which the rational course of action is to “maximize confidence in a good enough outcome even if the things go poorly” (instead of maximizing EUT); the concept is similar to “acceptable regret”^41^, ^42^ hypothesis of rational decision making, which postulates that we can rationally accept some losses without feeling regret.
Example: Annual screening mammography over 10 years in women older than 50 will prevent one death per 1000 from breast cancer but at cost of 50‐200 unnecessary false alarms and 2‐10 unnecessary breast removals.^43^ When it comes to decisions like these, which are value‐ and emotionally driven decisions, there are no right or wrong answers. Some women will accept harms for a small chance of avoiding death from breast cancer. Others may not.^44^
**Threshold model of rational action** proposes that the most rational decision is to prescribe treatment or order a diagnostic test when the expected treatment benefit outweighs its expected harms at a given probability of disease or clinical outcome.^45^ It has been formulated within EUT,^46^, ^47^ dual processing theories,^48^ and regret framework.^41^, ^42^, ^45^, ^49^
Example: See text and Figure 1.

**Table 2:** Core ingredients (“principles”) of rationality commonly identified across theoretical models^7^

P1: Most major theories of choice agree that rational decision making requires integrations of
• benefits (gains)
• harms (losses)
to fulfil our *goals* (eg, better health)
P2: It typically occurs under conditions of *uncertainty*
**•** rational approach requires reliable evidence to deal with the inherent uncertainties
**•** relies on cognitive processes that allow integration of probabilities/uncertainties
P3: Rational thinking should be informed by *human cognitive architecture*
**•** it is composed of type 1 reasoning processes, which characterizes “old mind” (affect‐based, intuitive, fast, and resource‐frugal) and type 2 processes (analytic and deliberative, consequentially‐driven, and effortful) of the “new mind”
P4: Rationality depends on the *context* and should respect epistemological, environmental, and computational *constraints* on human brains
P5: Rationality (in medicine) is closely linked to *ethics and morality* of our actions
**•** it requires consideration of *utilitarian* (society‐oriented), *duty‐bound* (individual‐oriented), and *rights‐based* (autonomy, “no decision about me, without me”) ethics

## 3 OVERUSE AND UNDERUSE UNDER NORMATIVE THEORIES OF DECISION MAKING

### 3.1 Evidence‐based medicine

Evidence‐based medicine (EBM) represents the dominant mode of clinical practice today. Evidence‐based medicine rationality rests on the link between taking action and believing what is true.^11^, ^12^, ^52^ That is, our actions and beliefs are justifiable (or reasonable/rational) as a function of the trustworthiness of the evidence (evidentialism) and the extent to which we believe that evidence is determined by credible processes (reliabilism).^11^, ^12^, ^52^ Evidence‐based medicine posits that when evidence is of higher quality (ie, it is *closer to the “truth”*), our estimates about benefits and harms are better calibrated.^12^ Under the premise that “rational people respect their evidence,”^11^ EBM postulates that recommending tests or treatments when there is high quality of evidence in favour of their support is the most rational recommendation to make. Indeed, there is some evidence that the probability that guidelines panels will issue strong recommendations (for or against interventions) is much higher when the quality of the evidence is actually better.^53^, ^54^ Thus, it appears that practitioners of EBM generally behave rationally. On the other hand, this EBM principle is not consistently followed: A study evaluating 456 recommendations made by 116 World Health Organization (WHO) guidelines panels found that about 55% of strong recommendations were based on low‐quality or very low‐quality evidence.^55^ People, including experts, are generally not skilled in distinguishing strong from weak evidence, an effect known as “meta‐cognitive myopia.”^56^ In most cases, following these recommendations would result in overuse, ie, an irrational policy according to the EBM rationality standard. However, a number of justifications could explain the WHO panel recommendations, and this may make them rational under different rationality theories (see below “Argumentative Theory of Reasoning/Rationality”).

An additional challenge for an EBM theory of rationality is that only about 20% of recommendations are based on consistent, high‐quality evidence.^57^, ^58^ In many cases, perhaps most, the recommendations cannot be made because of an absence of evidence. “Absence of evidence is, however, not evidence of absence”^59^—a lack of high‐quality evidence does not mean that the intervention is not effective. This creates situations ripe for both underuse and overuse. The latter occur when clinicians use their uncontrolled experience or “best judgment” in the absence of empirical data. However, most often, the major government or professional organizations are reluctant to recommend interventions for which there is no reliable evidence of its beneficial effects. Thus, rational behaviour according to EBM may lead primarily to underuse—denying health interventions to those who may need it.

### 3.2 Expected utility‐based decision analysis

Decision analysis is the second most commonly used normative theory in clinical medicine. It is typically used in cost‐effectiveness analyses as well as to guide development of guidelines for practice.^16^, ^60^ With respect to rationality, decision analysis is based on expected utility theory (EUT). According to EUT, when faced with several possible courses of actions, the rational decision is judged to be the one based on the selection of the alternative with highest expected utility—for instance, the one with the highest quality‐adjusted life years. Note that EUT is the only known theory of choice that satisfies all the mathematical axioms of rational decision making.^7^

One of the major advances in the field of decision making was the development of the so‐called threshold model.^45^, ^46^, ^47^ The *threshold* embodies a critical link between evidence (which exists on the continuum of credibility) and decision making (which is a categorical exercise—we decide to act or not act).^45^ The threshold model stipulates that the most rational decision in medicine is to initiate an intervention when the expected benefits outweigh its expected harms at a given probability of disease or clinical outcome (Figure 1A).^46^, ^47^ Figure 1A illustrates that, as the therapeutic benefit/harm ratio increases, the threshold probability at which treatment should be given is lowered.^46^, ^47^ Conversely, if a treatment's benefit/harm ratio is smaller, the threshold probability for therapeutic action will be higher.^46^, ^47^ For example, Basinga and colleagues estimated that benefit/harm ratio of administering antituberculosis therapy to a patient with suspected tuberculosis (TB) is about 36 in terms of morbidity/mortality outcomes.^50^ This converts into a low threshold probability of about 2.7%.^50^ Thus, according to EUT, rational physicians should prescribe drugs against TB when the probability of TB exceeds 2.7%.^50^ At the probability of 2.7%, this means that most patients suspected of having TB will actually not have TB. As a result, acting according to EUT, the normative theory widely accepted as the gold standard in medicine, will predictably lead to further increase (and resource waste) in the use of diagnostic and treatment interventions!^45^, ^49^, ^61^ This can hardly be considered a rational course of action. Note that because most evaluation of drug effects passes through the scrutiny of regulatory approval agencies such as FDA, they will be approved for use in practice only if the benefits outweigh the harms; similarly, most diagnostic tests are perceived as harmless. This means that overtesting and overtreatment are built into the EUT model. Underuse is also possible, but that usually occurs as a result of poorly calibrated prediction models that may (mis)estimate the probability of a disease/outcome to be below the threshold, when it is actually above the threshold. This is an epistemic (ie, knowledge‐related) issue caused by poor predictive evidence; it should be distinguished from the effects of emotions on estimates of probability and the consequences of decisions. As explained below, acting based on regret theory or a dual processing theory of rationality can modify an action threshold in a way that would appear more rational to a decision maker.

## 4 OVERUSE AND UNDERUSE UNDER DESCRIPTIVE THEORIES OF DECISION MAKING

### 4.1 Interactionist or Argumentative Theory of Reasoning and Rationality

The Interactionist or Argumentative Theory of Reasoning (ATR) proposes that people make decisions because they can find reasons to support them. People do not necessary favour the “best” decisions or decisions that satisfy some criterion of rationality, but decisions that are most (socially) acceptable, ie, those that can be most easily justified to oneself and others and are less at risk of being criticized.^23^, ^24^ The theory stipulates that reason and rational thinking has evolved with a primarily social function to justify oneself, to convince others to be believed, and to gain others' trust (Table 1).^23^, ^24^ From the ATR perspective, it is easy to explain why conflicts of interest—defined to exist “when professional judgment concerning a primary interest (such as patients' welfare or the validity of research) may be influenced by a secondary interest (such as financial gain or desire to avoid a lawsuit)”^62^—are pervasive in medicine and difficult to eradicate. From the perspective of those to whom conflicts of interest apply, such behaviour may be quite rational (if not necessarily moral).

Similarly, in explaining the reasons why their strong recommendations were based on low‐quality or very low‐quality evidence, the WHO panel members^55^ gave a number of reasons for issuing such recommendations (see above).^63^ A typical reason for offering treatment was given as “avoiding underuse,” based on a conviction that a treatment is beneficial despite the fact that the panel made explicit ratings of the evidence quality as low or very low.^63^ Such a reason directly contradicts EBM principles of rationality.^7^, ^64^ Another frequently given reason is a concern that policymakers responsible for funding decisions will ignore recommendations that are insufficiently “strong.” In addition, WHO panellists sometimes feel wedded to long‐established practices and feel uncomfortable issuing any but strong recommendations regarding such practices.^63^ Importantly, as predicted by ATR, the reasons given by the panel members are not meant to satisfy a specific criterion of rationality but to convince other panel members to “vote” in a similar way. Whether a particular recommendation would lead to overuse or underuse was rarely explicitly invoked.

### 4.2 Emotions and regret theory of rationality

People's decision making often relies on emotions and intuition. Our feelings influence the way we perceive and process risks; this is known as the “risk as feelings” phenomena.^65^ Emotions lead to different ways in which probabilities and consequences of our actions (utilities) are evaluated. Affect‐rich situations may lead to *probability neglect* in which people are sensitive only to the presence or absence of stimuli, and recognize outcomes only as being possible or not,^66^ while in affect‐poor contexts, probabilities tend to be evaluated without such distortions.^67^, ^68^ The “risk as feelings” phenomenon can influence the way physicians make their decisions. For example, Hemmerich et al^69^ studied physicians who experienced negative emotions, such as having a patient die during “watchful waiting” for a small abdominal aortic aneurysm. They found that such physicians' management of the subsequent patients would be significantly affected, to the point that they would accelerate the timing of surgery, even if this would contradict normative EBM guidelines. These physicians appear to aim to minimize their feelings of regret in the management of the next patient.^69^

Regret is a cognitive emotion, which we are motivated to regulate to achieve our desired goals; many of our decisions are driven by the desire to avoid regret and minimize (perceived) risks.^38^, ^39^ It has been argued that rational decision making is associated with regret‐averse decision processes,^17^, ^18^, ^34^ particularly if the beneficial aspects of regret regulation, such as learning and explicitly considering the consequences of decision making, are decoupled from the deleterious ones (eg, self‐blame and self‐reproach).^70^, ^71^ *Importantly, unlike normative models such as those based on decision analysis, regret takes context into account*. When regret was taken into account, the threshold for giving treatment to a patient suspected of having TB dramatically increased (from 2.7% to 20‐60%) (see Figure 1B).^50^ The threshold model can also be reformulated accounting for regret.^41^, ^42^, ^45^, ^72^ The model predicts drops in the threshold level if a decision maker regrets failing to benefit more than they regret causing unnecessary harms. This possibly can lead to more false‐positive decisions,^73^ resulting in overtreatment and overtesting as one would expect in the case of the management of an individual patient. Conversely, if regret of harms is felt to be higher than failing to benefit, the threshold will increase.^41^, ^42^, ^45^, ^72^ Under these circumstances, fewer false positive decisions would be made, but more false negative ones would be made, resulting in more undertreatment and underdiagnosis.^73^

### 4.3 Dual process theories of cognition and rationality

Principle no. 3 states that rational thinking should be informed by human cognitive architecture (Table 2). According to dual process theories, human cognition can be thought of as a function of 2 types of processes: type 1 processes, characterized as “old mind” (affect‐based, intuitive, fast, and resource‐frugal), and type 2, “new mind” processes (analytic, deliberative, consequential, and effortful).^9^, ^14^, ^74^ In the setting of dual‐processing architecture, it is important to realize that regret, as a cognitive emotion, is characterized by a counterfactual reasoning process: It operates imagining “what if” scenarios—we regret when we compare the actual outcome to what might have happened, but did not.^38^, ^39^ In this respect, regret *serves as a link between intuitive and effortful* processes, providing a mechanism for a dual‐process rationality model.^7^, ^75^ The threshold model, which links the key features of clinical medicine: evidence with decision making, has also been formulated within a framework of dual‐processing theories.^48^ An empirical study testing prediction according to EUT versus regret theory versus a dual‐processing threshold model showed that the model based on dual‐processing theory of decision making provided the best explanation for the observed results.^76^ This is likely because the model integrates regret, EUT, and a switch between 2 cognitive domains that can explain how a decision maker increases or decreases an action threshold as a function of interactions between type 1 and type 2 processes.^48^ For example, consistent with Hemmerich et al,^69^ the model postulates that a physician's threshold will go up, if his or her recent experience was coloured with emotions when he or she saw the next patient facing a similar decision. The threshold will go down if no emotion (including regret) had affected the physician's perception of benefits and harms of health interventions.^48^ Rationality, according to dual‐processing theories, needs to take into account both analytical and affect‐based reasoning.^77^ This might sound counter‐intuitive at first, in that popular culture often contrasts emotionalism with rationality; however, without emotion, we have no goals, and without goals, there is no rationality.^14^ It is the regulation of emotions, particularly regret, which represents one of the key ingredients of rational behaviour.^37^ Thus, according to dual‐processing theories of decision making, incoherence between type 1 and type 2 processes can disrupt optimal decision‐making, resulting in overuse or underuse as a function of their influence on the action threshold.^45^, ^49^ This, as explained, may happen when research evidence on benefits and harms implies one course of action (eg, treat at a lower probability of disease), but context, emotion, or recent experiences indicate a different course of action (eg, treat at a much higher probability of disease).^48^, ^69^

### 4.4 Theory of bounded rationality: Adaptive/ecological rationality

Medical encounters increasingly occur within the setting of the limited time and in the context of the ongoing information explosion.^78^ A typical clinical encounter is approximately 11 minutes long with less than 2 minutes available to search for reliable information, with interruptions occurring, on average, every 15 minutes.^78^ At the same time, more than 6 million articles are published in more than 20 000 biomedical journals every year,^79^ with MEDLINE alone containing over 22 million indexed citations from more than 5600 journals.^80^ In addition, 75 randomized clinical trials and 11 systematic reviews are published every day.^81^ This information explosion needs to be contrasted with the human brain's limited capacity for information processing, memory limitations, and relatively low storage capability.^78^ The theory of bounded rationality (which serves as the basis for principle no. 4, Table 2) posits that rationality depends on the context, and should respect epistemological, environmental, and computational constraints of human brains.^7^ Under the real‐life complexity of the health care system and the limitations of human information processing, rational behaviour relies on *satisficing* process (ie, finding a good enough solution)^7^, ^25^, ^78^ instead of *maximizing* (ie, finding the best possible solution).

*Satisficing* is sometimes structured via heuristics, which represent mechanisms for implementing bounded rationality.^27^ Heuristics are widely used in medical education, as popular “mental shortcuts,” “rules of thumb,” clinical pathways, and algorithms. The use of *heuristics* is defined as “a strategy that ignores part of the information, with the goal of making decisions more quickly, frugally, and/or accurately than more complex methods”^20^ and may sometimes outperform complex statistical models, in a phenomenon known as “less‐is‐more.”^21^

The principle behind satisficing is that there must be a point (threshold) at which obtaining more information or computation becomes overly detrimental and costly; the use of heuristics helps the decision maker stop searching before this threshold has been crossed. 21 In clinical medicine, it is often implemented via fast‐and‐frugal trees, highly effective, simple decision trees composed of sequentially ordered cues (tests) and binary (yes/no) decisions formulated via a series of if‐then statements.^82^ Fast‐and‐frugal trees can be linked to EUT and regret via the threshold model.^82^ A variant of *satisficing* known as “robust satisficing” is proposed to regulate regret,^17^, ^18^, ^37^ a concept similar to “acceptable regret”: We can rationally accept some losses without feeling regret.^41^, ^42^ “Acceptable regret” is shown to explain both underuse and overuse when compared against deviations from normative standards.^41^, ^42^ It explains why the “stubborn quest for diagnostic certainty,”^83^ that is, overtesting in the face of already sufficient evidence, widely considered to be one of the main culprits of increasing health care costs, may not be irrational.^41^, ^42^, ^61^, ^84^ For example, in an end‐of‐life setting, patients would accept a potentially wrong referral to hospice, only if the estimated probability of death within 6 months exceeded 96%. That is, they would accept hospice care without regretting it only if they are virtually certain that death is imminent.^84^ This may explain why dying patients are consistently referred to hospice very late, typically averaging less than 1 week before dying.^85^

### 4.5 Deontic introduction rationality: linking the rationality of “is” with the rationality of “ought”

We have consistently referred to the essence of clinical practice as the integration of empirical evidence with categorical actions (yes/no) that fundamentally defines decision making. In a medical context, rationality requires integration of evidence related to the problem at hand (“is”, which is derived from our observations) with the goals and values of decisions and (potential) actions (“ought*”*) (Principle Nos. 1 and 2, Table 2). It is these rationally guided *ought* decisions that allows us to achieve our goals. We referred to the threshold model of decision making as a model that serves as a link between evidence and decision making.^45^ Recently, deontic introduction theory was developed^29^, ^86^ to provide psychological mechanisms for how normative (a.k.a. “deontic”) rules for actions can be generated by linking empirical evidence to values and the transference of value from goals to actions.^29^, ^86^ Interestingly, there seems to be an evolutionary background for both the need for reliable evidence to help us function in our environment^87^, ^88^ and for the generation of normative “ought” or “should” rules (“Faced with the knowledge that there *are* hungry children in Somalia, we easily and naturally infer that we *ought* to donate to famine relief charities”).^29^ Physicians seem to generate deontic “ought” or “should” rules routinely. They first link evidence with outcomes to create explanations in terms of causation (“If you smoke, you will likely get lung cancer”). They then infer values from outcomes (“Lung cancer is an undesirable outcome”; therefore, “Smoking is bad”), which in turn results in value transference from goals to actions to create a normative conclusion (“You should not smoke”). The action rules thus created reflect pragmatic rationality that involves instrumental “oughts.” (Note that instrumental “oughts” should not be confused with evaluative “oughts,” which reflects overall value judgments such as moral judgments).^19^ The former are typically accurate *within a specific setting*, constituting of if‐then rules, while the latter *aim at universally valid statements* (even though they often cannot be separated from the context). So acting on normative conclusions frequently seen in oncology practice, such as “Given that the diagnosis of metastatic cancer *is* made, the patient *ought* to be treated with chemotherapy,” can be rational in one setting, but irrational in another. This can result either in overtreatment with futile therapy, as is often observed in the end‐of‐life setting,^89^ or undertreatment, as in cases when treatment is inappropriately denied based on an arbitrary (usually older) age or due to “excessively high” costs.

Deontic introduction theory emphasizes the crucial role of context in generating normative rules for guiding behaviour. Context defines the goals of the individual as well as their beliefs in how to cause them to materialize. According to *grounded rationality* 33 (Table 1), the rational course of action represents the action aimed to achieve our goals as a function of epistemic context—the evidence and knowledge available to us at the time of making such decisions. Such epistemic context is subject to cognitive variability, that is, the individual and cultural characteristics of the decision maker. For example, decisions on opting for palliative care are sensitive to cultural and individual values.^90^ This again serves to underline our thesis that no single theory of rationality can fit all decision makers in all contexts. What might be rational for a specific context may not be rational in a different context and might not even be rational for another provider in a similar medical context but from a different culture.

## 5 DISCUSSION

Rationality revolves around finding the most effective procedures to achieve our goals.^7^ As “no one size model fits all” clinical circumstances,^7^ these goals may be differently, but correctly, pursued and achieved under different theories of rationality. Purely normative models can often be off the mark, as they rely on mathematical abstractions, whereas prescriptive models of rationality^10^ aim to realize rational solutions by relying on the accumulated knowledge of human cognitive architecture and make recommendations accordingly. We propose that prescriptive rational models in clinical decision making should also make use of whichever model best fits a specific context. For example, operational achievements of the goals in health care can be realized by linking evidence with decisions via the threshold model.^45^ According to the threshold model, rational decision making consists of prescribing treatment or ordering a test when the benefits of treatment exceed its harms for given probability of disease or clinical outcome.^45^ However, thresholds can vary as a function of different contextual factors that play roles in some theories of decision making and not in others.^45^ As a result, what is defined as rational or irrational actions resulting in overuse or underuse is inextricably intertwined with whatever theoretical frameworks within which these decisions are considered. That is, rational behaviour under one theory may be irrational under a different viewpoint.

One of the fundamental challenges for medical decision making is that goals often conflict and that rational attempts to achieve one goal may prevent achieving another. According to Stanovich,^34^ rationality means achieving a coherence among goals, and we need to rely on both normative and descriptive procedures to coherently integrate across goals. This is known as meta‐rationality^34^—asking reflectively about the appropriateness of our emotional reactions to a decision. “The trick may be to value formal principles of rationality, but not to take them too seriously.”^34^

However, coherent integration of goals may sometimes be impossible. For example, we may have a rational goal to extend a patient's life, but resource use may exceed what is affordable. That is, decision makers often face a trade‐off between goals and interests of individuals and the wider society, pitching duty‐bound, deontological decisions against the utilitarian ethics. These goals are often expressed in terms of “value,” where “value” is defined as equivalent to clinical benefit/cost ratio. Formally, the most common metric to gauge the “right” value for health care is to calculate the incremental cost‐effectiveness ratio (ICER) among competing health interventions, ICER = (cost1 − cost2)/(effectiveness1 − effectiveness2). Typically, effectiveness is expressed as the quality life‐adjusted years gained for determining whether a given health intervention is considered beneficial. What is acceptable according to ICER depends on a particular society, which may decide not to offer a particular treatment or a diagnostic test if the societally agreed upon ICER threshold is exceeded. In the United States, for example, the generally acceptable ICER threshold is between $50 and $200 K per quality life‐adjusted year.^91^ In contrast, the WHO considers an intervention to be cost‐effective if the cost of the intervention per disability‐adjusted life year averted is less than 3 times the country's annual gross domestic product per capita.^92^ Using a different definition, and neglecting relevant context such as the disease burden and the available budget, may result in a paradoxical— and seemingly irrational—allocation of a country's health budget, as demonstrated by Marseille et al in their analysis of the WHO ICER thresholds.^92^ Fundamentally, all these initiatives expose tensions between societal vs individual interests.^73^ Interestingly, testing of the role of deontic introduction in moral inference found that the tendency to infer normative conclusions mostly coheres with utilitarian (rather than deontological) judgments,^86^ which can explain increasing outrage over ever‐increasing health care costs.^93^

Another example of a goal conflict, one that all too often leads to “overtreatment” with aggressive therapy rather than a more appropriate palliative approach such as hospice, is the end‐of‐life setting. The usual goal for medical decision making is to improve health, but what are appropriate goals at the end‐of‐life? A “good death” is not “better health” in the usual sense, yet it can be rationally accepted, most importantly at the level of intrinsic emotional peace. A theoretic approach to rational decision making must accommodate this goal. Here again, invoking regret may prove the mechanism allowing the peaceful exit that many humans typically desire. Consistent with Aristotle's “dead bed test” of no regret—life lived with no unfulfilled potentials weighing on our souls—it was found that elicitation of regret can actually improve decision making at the end of life.^40^ Thus, rational decision making has to take into account both analytical and affect‐based reasoning.

A “unifying theory of rationality” is likely not possible, particularly because decision making is extremely context sensitive (and, as explained, normative theories typically fail to take context into account).^7^ We also believe that context setting is a prerequisite to a rational approach to both practical and theoretical considerations to problem‐solving and decision making. How a given clinical problem should be approached is ultimately an empirical question. By calling for “normative pluralism” and pragmatic rationality, according to which the context and the clinical situation should be matched to a contextually appropriate theory of rationality, we believe that the current unsatisfactory situation in health care could dramatically improve. Although this position incorporates an element of relativism, by acknowledging contextual dependence, it is a moderate type of relativism rather than a stronger “anything goes” version.^94^, ^95^

Our paper also has important practical implications. Physicians are increasingly paid according to the quality of care they deliver and penalized for overuse/underuse.^96^ What our analysis shows is that these policy initiatives cannot be devoid of the theoretical rationality framework in which quality improvement assessments operate. Financing of health care services, which is increasingly being proposed to be a function of the measurement of overuse and underuse of health services, should be determined based on the choice of theoretical framework.

We believe that an attempt to define theoretical framework(s) to measure appropriateness of care is what is largely missing in the current discussion of overuse and underuse of care. Even though both overuse and underuse are widely acknowledged as an empirical phenomena in modern health care,^51^, ^97^, ^98^, ^99^, ^100^ actual measurement of overuse and underuse has been difficult to achieve.^97^, ^98^

Two methodological approaches have dominated measurements of overuse and underuse: (1) comparing the use of health care services against some sort of predefined “truth” or “gold standard” and (2) detecting unexpectedly wide variations in the delivery of health care services.^97^, ^98^ The first approach relies on a “correspondence theory of truth,” which assumes that there is “objective reality” and that “truth” is based on the correspondence of ideas, concepts, and theories with facts.^101^, ^102^ This typically takes a form of measuring outcomes against evidence‐based guidelines. However, as discussed above, evidence is often challenged and finding an incontrovertible “truth” that is uniformly accepted is, in practice, extremely difficult, perhaps impossible. One possible solution is by seeking correspondence with the *goals* of the decision maker (rather than with an “objective truth” outside the decision maker), as argued above.

The second approach, which measures overuse and underuse by assessing (surprisingly wide) variations in care, relies on the “coherence theory of truth,” according to which statements or judgments are “true” if they cohere with other judgments or statements.^101^, ^102^ Thus, it is assumed that similar patients in similar conditions in similar settings should be managed similarly, with minimal variation. This approach is typically based on the analysis of practice patterns from large data sets^33^, ^90^ and fundamentally disregards individual patients' circumstances and their values and preferences. For example, use of more medicalized terminology in discussions with patients often leads to more aggressive treatment and overuse.^103^ In this sense, coherence is often used to define “rationality”—given the premises, certain conclusions should be necessarily drawn if a rational reasoning process is followed. However, deductive argument validity only guarantees rational conclusions given rational premises, which is not necessarily always the case. If the premises are false, even if the reasoning is valid, the conclusion will also be false. In contrast, correspondence is concerned with the accuracy of judgments or claims against some criterion of accuracy,^101^, ^102^ which makes it potentially more useful for rationality in clinical decision making.

Ultimately, both measurement and mitigations of overuse and underuse will not improve until they are placed within a better framework of rationality theories.^101^, ^102^ Although there are many theories of truth, broadly speaking, normative theories of rationality reflect the coherence theory of truth, while descriptive theories of rationality tend to rely on the correspondence theory of truth. As we stress above, different medical problems will require different theoretical approaches. In our view, further advances in health care, including reducing the rates of overuse and underuse, will only be possible with an explicit identification of the theoretical framework from which the problem is addressed.
